# New Evidence on Variations of Human Body Burden of Methylmercury from Fish Consumption

**DOI:** 10.1289/ehp.7857

**Published:** 2006-01-18

**Authors:** René Canuel, Sylvie Boucher de Grosbois, Laura Atikessé, Marc Lucotte, Paul Arp, Charles Ritchie, Donna Mergler, Hing Man Chan, Marc Amyot, Robin Anderson

**Affiliations:** 1 Institute of Environmental Sciences and; 2 CINBIOSE, Collaborative Mercury Research Network, Université du Québec à Montréal, Montréal, Québec, Canada; 3 Faculty of Forestry and Environmental Management, Collaborative Mercury Research Network, University of New Brunswick in Fredericton, Fredericton, New Brunswick, Canada; 4 Center for Indigenous People’s Nutrition and Environment, Collaborative Mercury Research Network, McGill University, Ste-Anne-de-Bellevue, Québec, Canada; 5 Department of Biology, Collaborative Mercury Research Network, Université de Montréal, Montréal, Québec, Canada; 6 Department of Fisheries and Oceans Canada, Collaborative Mercury Research Network, St. Johns, Newfoundland, Canada

**Keywords:** First Nations communities, fish consumption, hair mercury levels, human metabolism, methylmercury

## Abstract

Epidemiologic studies commonly use mercury (Hg) level in hair as a valid proxy to estimate human exposure to methylmercury (MeHg) through fish consumption. This study presents the results yielded by a complete data set on fish consumption habits, Hg levels in edible fish resources, and corresponding Hg accumulation in hair, gathered in three distinct communities of eastern Canada. For one of these communities, the average hair Hg concentration was 14 times less than the expected value based on calculated daily oral exposure and current knowledge of MeHg metabolism. This finding could be explained by differences in specific genetic characteristics and/or interactive effects of other dietary components.

Fish constitutes the main dietary protein source for many populations worldwide. However, the presence of methylmercury (MeHg) in fish flesh can affect the health of frequent fish consumers. Health risk assessment requires fitting the level of exposure of food-borne contaminants to a mathematical function relating exposure to effects on health (Cronin et al. 1993). Most epidemiologic studies dealing with the MeHg issue use mercury (Hg) levels in hair as the only indicator of human exposure, without relating this signal to actual fish consumption patterns among populations. The expected constant and linear relation between MeHg oral dose and body burden is used by government officials to establish guidelines on safe levels of MeHg exposure. Our objective in the present study was to test the validity of this approach by relating measured exposure to MeHg through fish consumption and associated measured levels of Hg in human hair. Using information yielded by *a*) a complete data set that includes, for three distinct Canadian communities, fish consumption patterns, biomarkers of human exposure, and related levels of Hg in edible fish species; and *b*) reanalysis of other published research related to fish consumption and hair Hg concentrations, allowed us to demonstrate that Hg levels in hair do not always reflect the reported level of MeHg intake via fish consumption, and therefore, the expected constant and linear relation between MeHg oral dose and body burden seems to vary significantly among ethnic groups. This observation could lead to questioning of regulatory policies and advisory guidelines on fish consumption.

## Materials and Methods

This study was conducted in 2002 during the course of a broader investigation on the behavior and fate of Hg in contrasted environments, supported by the Collaborative Mercury Research Network (COMERN). Sports and subsistence fishers of the Abitibi (*n* = 146) and Lake St. Pierre (*n* = 130) regions (Quebec, Canada), mainly of Caucasian origin, and the First Nations people of the Innu community (Sheshatshiu, Labrador, Canada; *n* = 118) were surveyed about their dietary habits for the 3-month spring season. This period corresponds to the “camp” season for the Innu, when they partake in the traditional way of life in remote hunting and fishing settlements, subsisting solely on the country food they collect, without any external supplies from market sources. Unless otherwise specified, fish specimens used to characterize the communities’ exposure to MeHg were collected with the help of local fishermen in the same lakes they use for either subsistence or leisure fishing. We collected hair samples to evaluate MeHg body burden. We gave this biomarker priority because it allows sequential analysis and because it allows integration of information based on a longer period. Blood Hg content would reflect only recent Hg intake.

### Mercury levels in fish.

Total Hg levels in fish were evaluated at the University of Quebec in Montreal (Quebec, Canada; UQAM) and at the Department of Fisheries and Oceans (St. Johns, Canada). These laboratories undergo twice-yearly interlaboratory calibration rounds administered by the Mercury Quality Assurance Program of the Canadian Food Inspection Agency, with analytical variability systematically lower than 10%. A correction factor of 0.85 was applied to all measurements to account for the proportion of organic versus total Hg.

A polynomial regression (Tremblay et al. 1998) was applied to normalize the relationship between fish length and Hg levels in fish tissues. This procedure is powerful enough to fit either linear or nonlinear regressions between the two variables for the different cohorts. MeHg levels used to calculate exposure were determined from the polynomial regressions at a standardized length that correspond to the size of fish (species dependent) regionally consumed by the participants, according to the dietary assessments.

### Dietary assessment and exposure to MeHg.

Detailed information on yearly and seasonal fish consumption patterns were obtained using a semiquantitative food frequency questionnaire (FFQ) developed specifically for each community. Questions about specific fish species consumed were incorporated into the questionnaires devoted to the different communities. The questionnaires were pretested with community members to evaluate content validity. Other information, including fish species and their origins (fishing location, market fish, canned products), were obtained.

The “fish frequency” was then calculated as the number of meals for each specific fish species. Each specific species frequency was multiplied by the portion consumed (in grams) that was indicated by the participant. Examples of portion were provided, and the equivalence in grams was derived by the research team. In addition, local fish species were collected, Hg levels were measured, and mean daily Hg intakes through fish consumption were estimated. Individual Hg intake through fish consumption were estimated adding all frequencies × portion (grams) × fish Hg levels for each fish species.

### Hair sampling and analyses.

Hair samples were collected from participants, and Hg concentrations were measured using a standardized protocol (Gill et al. 2002). Because hair grows approximately 1 cm per month, they were cut into 1-cm-long samples and analyzed independently to establish the Hg content. Hair strands from the root were taken from the occipital region and then placed in plastic bag with the root end stapled. The 3 cm of hair corresponding to the 3 months described in the dietary assessments were analyzed at the Laboratory Services of the First Nations and Inuit Health Branch of Health Canada (Environmental Research Division) and at UQAM. Analyses of total and inorganic Hg were performed according to the method described in Farant et al. (1981). Analytical quality control was ensured with standard hair samples, provided by the Hair Mercury Interlaboratory Comparison Program of Health Canada. Both laboratories participate twice each year in the interlaboratory calibration program Hair Mercury Proficiency Testing Program of Health Canada, with analytical variability systematically lower than 10% (Gill et al. 2002).

### Simulation runs.

Starting with the extensive quantitative analysis made by the National Research Council (NRC 2000) on Hg toxicology and metabolic dose response that became the main reference in this field of Hg science, we assembled a graphic interfaced STELLA-based one-compartment model (ISEE Systems, Lebanon, NH, USA) that offers a convenient way to test the relationships of various data sets on human MeHg exposure presented by the NRC. It provides for this study a simple and practical tool to compare the relative response of groups to certain levels of Hg exposure rather than an absolute prediction method. The well-established toxicokinetics parameters used by the model are presented in Table 1 (NRC 2000; Rice et al. 2003; U.S. Environmental Protection Agency 1997). We successfully tested and validated this particular rendering of the NRC’s work against experimental data sets and other existing models on human response to MeHg exposure (Birke et al. 1972; Carrier et al. 2001; Kershaw et al. 1980; Sherlock et al. 1984). This tool was used to assess and compare our experimental data sets of the ratios of dose (or exposure to MeHg) to response (or hair Hg levels) of the different communities involved in this study with the values that could be expected from the widely recognized knowledge on Hg metabolic behavior and rates yielded by the model’s outputs.

## Results

Table 2 presents the calculated daily exposure for the 3-month spring season for the three populations as well as a comparison between the simulated and mean measured Hg hair values corresponding to the same period. There is close agreement between the measured Hg levels in hair and estimated values from the model in Abitibi and the Lake St. Pierre region (estimated average within the range of the standard deviation of hair measurements). However, in Labrador, the estimated average hair Hg concentration was > 14-fold the measured value. Furthermore, the ratios of mean measured hair Hg level to calculated daily exposure differ by a factor of > 10 between the Innu and the Lake St. Pierre cohort and a factor of 7 between the Innu and the Abitibi cohort, which contradicts the constant relation between exposure and hair Hg levels expected according to accepted theories.

To confirm this observation, we gathered and reanalyzed published but separate data sets of Hg levels in hair in different communities, and then information on their dietary habits and plausible sources and levels of Hg exposure. Only data sets that contain information sufficient to build reasonable scenarios on fish consumption patterns and contamination were included. Results of simulation runs on measured and estimated Hg levels in hair for these populations are presented in Table 3.

One of the most complete data sets of hair Hg concentrations and fish consumption patterns is reported by Lebel et al. (1997) and Dolbec et al. (2001), who documented the behavior of riparian populations living along the shores of the Tapajòs River in the middle of the Brazilian Amazon. Hair Hg signal simulated by the model using their field data is consistent with the observed field hair Hg levels and within 100% of the measured concentration.

However, estimated hair Hg levels for the different aboriginal people communities of Canada (Nunavik, Eastmain, and White Dog) presented in Table 3 were about 500% higher than the measured concentrations. Interestingly, similar variation was observed using data sets published by Yasutake et al. (2004) and obtained in different populations in Japan (Table 3). Nakagawa et al. (1997) also reported a mean Hg intake of 170 μg Hg/week from fish and shellfish for the Japanese population, which would correspond, using our model, to a Hg hair signal of 6.7 ppm (compared with an average measurement of 1.8 ppm; Yasutake et al. 2004).

## Discussion

In this study, using validated tools and procedures, we found that for the Innu community, Hg levels in hair did not reflect the reported level of MeHg intake via fish consumption. The discrepancies between the measured Hg levels in biomarkers and the modeled Hg levels could be questioned considering that many sources of variability, including instrumental and reporting bias, may have been introduced into the design of the study and/or analysis of the results.

### Analytical variability and determination of fish MeHg levels.

Based on interlaboratory calibration rounds administered by the Mercury Quality Assurance Program of the Canadian Food Inspection Agency, and on the Hair Hg Proficiency Testing Program of Health Canada, analytical variability related to the Hg quantification in fish and in hair samples is < 10%, which leads to a quite accurate evaluation of Hg content in fish and in hair samples. All hair Hg measurements were performed on representative samples, using recognized analytical techniques (Gill et al. 2002). Fish Hg data used to estimate exposure levels are mainly either from direct field measurements in the lakes harvested by the communities to minimize interlake variability or from otherwise well-documented sources. Furthermore, we elected to normalize the fish Hg levels through a polynomial regression between fish length and Hg levels to *a*) compensate for fluctuations of fish Hg levels with length, with the bigger fish usually having higher Hg levels; *b*) account for intralake variability of fish Hg levels for a given species; and *c*) extrapolate specific fish Hg levels at edible length as reported in the regional dietary assessments. To our knowledge, the estimates calculated using this protocol remain among the most accurate in the available population-scale studies.

### Variability related to exposure assessment.

In Labrador the period sampled corresponds to the “spring camp” season for the Innus. For this 3-month period they live isolated in remote hunting and fishing settlements, without external food supplies and subsisting solely on their fish and wildlife harvest. Dietary information for the Innus was gathered just after this period, reducing recall bias. In this community no advisory promoting fish consumption was under way. Therefore, social and/or cultural pressure to report traditional food habits could not account for a potential overreporting bias. Detailed information was gathered to provide an exhaustive dietary profile and a good evaluation of fish Hg content based on actual fish species lengths and fishing sites reported by the participants.

Dietary survey, which may include reporting bias of participants, would result in over-or underestimation of intake. Variability in self-reported dietary intake threatens inferences from studies relying on instruments such as FFQs. Several authors have addressed the issues of reliability and validity of FFQ as estimators of dietary patterns (Horner et al. 2002; Hu et al. 1999; Kipnis et al. 2002; Shatenstein et al. 1999). Studying the reliability and relative validity of fish consumption data obtained in an exposure assessment study among sport fishers in the Montreal area, Shatenstein et al. (1999) conclude that the FFQ provides a reliable and relatively accurate indication of sport-fisher fishing practices, species selection, and sport-fish consumption habits. In this study ascertainment of the consistency of estimated portion sizes and number of fish meals was performed during a two-season exercise over 2 years. Elsewhere, studying the reproducibility and validity of dietary patterns assessed with an FFQ, Hu et al. (1999) found good reliability correlation coefficients (0.70). Validity analysis, comparing FFQs with diet records (gold standard), showed moderate to good correlation coefficients for fish and other seafood (0.51–0.74). The authors concluded that the major dietary patterns derived from the FFQ have reasonable reproducibility and validity. However, only men were included in their sample. A study conducted in a group of post-menopausal women also concluded reliable and accurate measure of usual intake of major nutrients and food groups among women in Shanghai (Xu et al. 2004). In a study designed to evaluate the reliability and validity of an FFQ for low-income Mexican Americans, McPherson et al. (1995) found correlation coefficients ranging from 0.61 to 0.77, comparing results obtained from FFQ with 3-day food records. Reliability correlation coefficients calculated after a 2-month interval ranged from 0.85 to 0.90. These high correlation coefficients were explained by the authors as the lack of diversity in the diets of the participants. However, different results were found by Horner et al. (2002) and Kipnis et al. (2002). In a postmenopausal study (*n* = 102), Horner et al. (2002) showed that underreporting appeared to be relatively high. However, this variation seemed to be explained mainly by participant characteristics, and evidence suggested more underreporting among women who were younger or had high social desirability score. Kipnis et al. (2002), in a study aimed to evaluate measurement error structure in dietary assessment instruments using urinary nitrogen excretion as a reference biomarker for protein intake, showed overestimation (up to 230%) and underestimation (up to 240%) of FFQ. However, even in this worse-case scenario, such variability would not explain the 14-fold difference observed in our study between the observed Hg level in hair and the expected values.

### NRC model.

Intrapopulation variability of human metabolic handling of MeHg has been reported in most epidemiologic studies addressing the issue. Pharmacokinetic constants were reported to vary extensively between individuals and between groups. This variability yielded the greatest uncertainties for its predictions. For example, the ratio used by the NRC between Hg content of hair versus blood is 250 but ranged elsewhere from 140 to 370 (Bartlett et al. 2000; World Health Organization 2003), or roughly +48%/–45%. Likewise, the generally recognized value for whole-body half-life of organic Hg is 70 days (also used by NRC), but this number is reported to vary between 44 and 80 days (Willes 1977; World Health Organization 2003), or roughly +15%/–63%. This variability is taken into account by existing norms through the inclusion of uncertainties factors used by risk assessment models to limit specific risks that could emerge from individuals’ divergence from averaged dose–response ratio. However, although intrapopulation heterogeneity makes consensus, it is to our knowledge the first time that a homogeneous divergent signal among a population such as the one measured in the Innu community is reported. This discrepancy is significant and cannot be accounted for by the model’s variability, even if considered as rather improbable best-case scenarios (half-life, 44 days; hair:blood ratio, 370) or worst-case (half-life, 80 days; hair:blood ratio, 140).

### Other simulations.

Finally, the scenarios describing consumption frequencies for the Inuit and Cree communities reported in Table 3 were established taking into account both published and traditional knowledge of communities’ eating habits, as well as the presence of alternate food sources that could replace local fish resources, and carry biases on our estimates. Both the Inuit and Cree communities live in remote, secluded locations and rely heavily on subsistence living. The Inuits exhibit opportunistic feeding habits, based on occasional availability of food sources, for example, frequently feeding on mammals (seals, whales) for extended periods, after fruitful hunts. On the other hand, the Cree study was performed in 1988, which corresponds to the climax period for both the exploitation of the newly impounded reservoirs from the La Grande river complex by the community and peak contamination of the reservoirs’ fish resource by Hg (Schetagne and Verdon 1999). For these reasons we feel that the above estimates are realistic.

## Conclusion

Considering that reanalysis of published data are presented solely in the context of supporting strong evidence from field data, we suggest that, contrary to well-received, accepted, and commonly used scientific precepts, the relation between MeHg oral dose and body burden—expressed as human MeHg exposure through fish consumption versus Hg levels in hair—may vary among certain ethnic groups. Several possible hypotheses are proposed to explain this observation.

The nature (density, molecular structure, growth rate) of hair might differ between ethnic groups. However, we could not observe any statistical difference in hair weight per centimeter between the Labrador, Lake St. Pierre, and Abitibi cohorts. Other observations (Wolfram 2003) also suggest that hair molecular structure should not differ to a great extent between the groups.

Mercury metabolic excretion rates might vary according to ethnicity. It is well recognized that Japanese, Vietnamese, Chinese, and First Nations populations, which are of Asian ancestry, experience a genetic polymorphism for enzymes ALDH2 and glutathione *S-*tranferase involved in ethanol metabolic excretion and Hg export from human cells (Strange et al. 2000; Walsh et al. 2001; Yokoyama et al. 2002). These genetic polymorphisms could affect elimination rate. Animal studies provide evidence for a major role of γ-glutamyl transpeptidase (GGT) in regulating the tissue distribution and elimination of MeHg in GGT-deficient mice (Ballatori et al. 1998). Differences in enzymatic expression might result in differential metabolic process of MeHg. Currently, no study has been designed to address those interactions in human populations. Therefore, the extent to which interpopulation variability in biomarkers at similar doses is attributable to genetic differences in susceptibility remains unknown.

Combined effects of specific metabolic processes or specific components present in the diet of the above populations after particular traditional food consumption and/or cooking habits, might decrease the metabolic absorption or increase the excretion of MeHg. If proven, this hypothesis would culminate in reduced health risks associated with fish consumption.

Regardless of the factors explaining the above observations, the impact of the evidence presented here on normative policies regarding safe levels of MeHg exposure is obvious. The current regulation guidelines used by both national and international agencies are based on the assumption that hair Hg concentration is a valid indicator of oral dose and toxic end points for all populations. If this assumption is proven invalid and as suggested by our findings, the actual guidelines must be fish consumption do not fully reflect the true metabolic response of different ethnic communities to the presence of Hg in their food source, and fish consumption advisory guidelines have to be redrafted using information collected specifically from local communities. Considering the importance of fish as a protein source for many First Nations communities and/or subsistence fishers, further assessment is needed to properly inform consumers frequently enjoying fish meals about the health costs–benefits related to fish consumption.

## Figures and Tables

**Table 1:** Pharmacokinetic parameters used in the STELLA model.

Parameter	Value
Half-life of Hg in body	70
Maximum Hg input from tooth amalgam (μg/day)	21
No. of teeth with amalgam fillings (maximum = 32)	12
Body absorption rate of inorganic Hg^2+^	0.15
Body absorption rate of inorganic Hg^0^ (μg/day)	0.03
Hg accumulation in the liver	70%
Hg accumulation in the brain	10%
Ratio of hair-to-blood Hg levels	250
Rate of hair growth (cm/day)	1.1
Hair to emerge from scalp (days)	20

Data from NRC (2000).

**Table 2:** Simulation runs for the three regions under study.

Region	Calculated daily exposure (μg Hg/day/kg bw)^a^	Measured Hg levels in hair (ppm; first 3 cm)	Ratio between Hg hair levels and calculated exposure	Modeled Hg levels in hair using exposure (ppm)
Lake St. Pierre (*n* = 130)	0.068 (0.109)^b^ (0.018)^d^	0.83 (0.97)^c^ (0.17)^d^	12.2	1.2 (0.33)^d^
Abitibi (*n* = 146)	0.139 (0.183)^b^ (0.30)^d^	1.2 (1.40)^c^ (0.23)^d^	8.6	2.3 (0.49)^d^
Labrador (*n* = 118)	0.342 (0.242)^b^ (0.044)^d^	0.4 (0.36)^c^ (0.065)^d^	1.2	5.7 (0.73)^d^

bw, body weight

a Measurements were performed on Hg levels in local species at standardized edible length, and canned tuna. Mercury data for market fish are from Dabeka et al. (2003).

b SD on average values, calculated from the variability associated with individual numbers of fish meals per species for the sampling season; an additional factor of 10% can be considered to account for the analytical uncertainty related to fish Hg level determinations.

c SD on the general mean of individual averaged hair Hg levels for the 3 cm corresponding to the dietary assessments; an additional factor of 10% can be considered to account for the analytical uncertainty related to hair Hg level determinations.

d 95% confidence limit (Colton 1974).

**Table 3:** Simulation runs for other populations.

Population	Estimated Hg level in local fish (ppm)	Average daily Hg intake (μg Hg/day/kg bw)^a^	Modeled Hg level in hair (ppm)	Measured Hg level in hair (ppm)	Variability (%)
Tapajos^b^ Brazil, 1996 (*n* = 36 women)	0.2	0.50	8.5	Median, 12.5	68
Nunavik^c^ Canada, First Nations Inuit, 1992 (*n* = 492)	0.5	1.07	18.1	3.8	476
Eastmain^d^ Canada, First Nations Cree, 1988 (*n* = 144)	1.1	1.37	23.4	50th percentile > detection limit of 2.5, 95th percentile > 6	~ 400
White Dog^e^ Canada, First Nations Ojibwa, 2003 (*n* = 48)	NA	1.53	25.8	4.5	573
Japan, f Miyagi district, 1999–2002 (*n* = 1,185)	0.5	0.80	13.5	2.3	586
Japan,^f^ Okinawa district, 1999–2002 (*n* = 1,019)	0.5	0.35	5.9	1.6	369
Japan,^f^ 10 districts, 1999–2002 (*n* = 8,665)	0.5	0.49	8.3	1.8	461

a Body weight (bw), 60 kg for Japanese and Brazilians; others, 70 kg.

b Calculated from field data on consumed fish species (Lebel et al. 1997) and Hg levels in fish (Dolbec et al. 2001).

c Mean fish Hg level reasonably set to 0.5 ppm, according to levels of contamination of food traditionally consumed by Inuits (Wagemann et al. 1998) and consumption frequency (Dewailly et al. 2001a): seal liver, 19 ppm; seal muscle, 0.6 ppm; arctic char, frequently > 0.5 ppm; lake trout, frequently > 0.5 ppm; whitefish, frequently > 0.5 ppm; white whale skin, > 0.5 ppm; beluga muscle, 1.04 ppm; beluga liver, 10.1 ppm. Blood measurement in Dewailly et al. (2001b) converted in hair signal using a hair:blood ratio of 250 (Schwartz 1999).

d Estimated according to Cree consumption habits: 20% of total diet from fish (Hydro-Quebec 2001); proposed diet (Hg levels in Dumont et al. 1998): one-fifth each whitefish from LaGrande 2 reservoir (Quebec), 0.5 ppm; whitefish, Eastmain river, 0.4 ppm; pike, LaGrande 2 reservoir, 3 ppm; pike, Eastmain river, 0.8 ppm; white sucker, LaGrande 2 reservoir, 0.8 ppm. Hair Hg levels are from Schetagne and Verdon (1999).

e Calculated from data set on consumption frequency and Hg levels in fish (Chan LHM, personal communication).

f Consumption frequency and Hg hair measurements are from Yasutake et al. (2004) and Nakagawa et al. (1997); mean fish Hg level reasonably set to 0.5 ppm, considering frequency of tuna consumption and type of tuna consumed, leading to an estimated mean tuna Hg level of 1.1 ppm.
